# Patient-reported assessment of medical care for chronic inflammatory skin diseases: an enterprise-based survey

**DOI:** 10.3389/fmed.2024.1384055

**Published:** 2024-04-18

**Authors:** Kerstin Wolk, Maximilian Schielein, Julia-Tatjana Maul, Fontaine Widmayer, Kerstin Wanke, Wolfgang Fischmann, Petra Nathan, Robert Sabat

**Affiliations:** ^1^Psoriasis Research and Treatment Center, Charité – Universitätsmedizin Berlin, Corporate Member of Freie Universität Berlin and Humboldt-Universität zu Berlin, Berlin, Germany; ^2^Interdisciplinary Group Molecular Immunopathology, Dermatology/Medical Immunology, Charité – Universitätsmedizin Berlin, Corporate Member of Freie Universität Berlin and Humboldt-Universität zu Berlin, Berlin, Germany; ^3^Novartis Pharma GmbH, Nurnberg, Germany; ^4^Department of Dermatology, University Hospital Zurich, Zurich, Switzerland; ^5^Faculty of Medicine, University of Zurich, Zurich, Switzerland; ^6^Novartis Pharma AG, Basel, Switzerland; ^7^BMQ Evaluation & Consulting GbR, Erlangen, Germany

**Keywords:** skin inflammation, atopic dermatitis, psoriasis, chronic spontaneous urticaria, hidradenitis suppurativa, quality of life, work productivity, biologics

## Abstract

**Background:**

Chronic inflammatory skin diseases (CISDs) are among the most common diseases in the Western world. Current estimates of medical care for CISDs are primarily based on surveys among patients in medical care facilities and on health insurance data.

**Aim:**

Survey-based examination to what extent CISD patients in health-aware environment consider their skin disease to be controlled.

**Methods:**

The survey of CISD patients was carried out in 2022 among the employees of a pharmaceutical company located in Germany and Switzerland. Software-based, anonymous, self-reported questionnaires were used.

**Results:**

The number of employees, who answered the questionnaire, was 905. Of these, 222 participants (24.5%) reported having at least one CISD. 28.7% of participants with CISD described their disease as being hardly or not controlled. Regarding the nature of disease, more than one third of participants suffering from hidradenitis suppurativa (HS) or psoriasis fell into the hardly/not controlled category. In contrast, the largest proportion of participants with chronic spontaneous urticaria (43%) or atopic dermatitis (42%) considered their CISD to be completely or well controlled. Only 35.5% of CISD sufferers stated that they were currently under medical care for their skin condition. Being under medical care, however, had no influence on the extent CISD sufferers considered their skin disease to be controlled. The number of active CISD episodes but not the total number of symptomatic days per year was negatively associated with poor disease control (*p* = 0.042 and *p* = 0.856, respectively). Poor disease control had a negative effect on the personal and professional lives of those affected, as deduced from its positive association with the extent of daily activity impairment and presenteeism (*p* = 0.005 and *p* = 0.005, respectively). Moreover, 41.4 and 20.7% of participants with hardly/not controlled disease stated that their CISD had a moderate and severe or very severe impact on their overall lives (*p* < 0.001), respectively. A severe or very severe impact of their CISD on their overall life was most commonly reported by participants with HS.

**Conclusion:**

Medical care for CISDs, even in an environment with high socio-economic standard and high health-awareness, still appears to be limited and has a negative impact on individuals and society.

## 1 Introduction

Chronic inflammatory skin diseases (CISDs) are among the most common diseases in the Western world. A recent survey conducted in a representative sample of the general population in 27 European countries estimated that ~25% of people aged ≥18 years suffered from CISDs during the previous 12 months (1). Atopic dermatitis (AD; 5.5%), psoriasis (PSO; 3.9%), chronic urticaria (1.0%), and hidradenitis suppurativa (HS; 0.6%) were among the most prevalent CISDs. The pathomechanisms and, consequently, the clinical pictures greatly vary among the CISDs (2). Impairment of the epidermal barrier function, Th2 cells (dominating the acute disease stage), and Th2/Th1/Th22 cells (dominating the chronic disease stage) play major roles in the pathogenesis of AD (3–5). Skin lesions of patients with AD are highly itchy and vary with disease stage and patient’s age. In acute stage, they are characterized by not clearly demarcated erythema with vesicles, excoriations, and serous exudate (4). In chronic stage, skin lesions are less erythematous, dry (xerosis), and thickened (lichenified) (4). The face and trunk are preferentially involved in small children while the flexor sides of the extremities are preferentially concerned in adults (4). In PSO, a Th17/Th22-cell mediated CISD, skin alterations in most patients manifest as sharply demarcated, erythematous plaques with silvery scales, preferentially located at the extensor sides of the extremities (6–9). Chronic urticaria manifests itself as itchy wheals and angioedema that occur spontaneously or as the result of trigger factors and that are primarily mediated by mast cell mediators (10–12). Skin alterations in HS develop in intertriginous sites in the form of deep-seated, painful nodules, abscesses, and pus-draining tunnels (13). The complex pathomechanisms in these patients include roles of neutrophils, Th1 and Th17 cells, and B cells (14–16). Not surprisingly then that due to the external visibility of the skin alterations and the association with itching, pain, scaling, exudation and/or infections, patients with those CISDs are not only physically but also psychologically burdened. If left untreated, CISDs lead to profound reduction in the quality of life (17, 18). Furthermore, patients with CISDs frequently suffer from systemic comorbidities (13, 19–21) that are associated with shortened life expectancy (22, 23). Owing to a substantial decrease in work ability and productivity of those affected, CISDs also have a significant negative socioeconomic dimension. In fact, HS alone leads to a 13 billion loss in gross value added in Germany (24). On the other hand, with the approval of biologics and high-efficacy oral compounds targeting pathogenetic immune mediators, the possibility of successfully treating many of these diseases has increased significantly over the past two decades (7, 25–27). Moreover, many European countries spend over a tenth of their gross domestic product on healthcare. This raises the question of how satisfied CSID patients are with the current care for their disease. In this study we performed a survey-based investigation to what extent CISD patients consider their skin disease to be controlled. The survey was carried out in a health-aware environment, namely among the employees of a pharmaceutical company, which itself has a high level of expertise in the field of CISDs. With Germany and Switzerland, the survey was conducted at two company locations with a high standard of medical care and with comparatively high levels of income.

## 2 Methods

### 2.1 Study populations

This was a prospective anonymous survey conducted among the employees of Novartis Pharma at the company locations Nurnberg (Germany), Rotkreuz (Switzerland), and Basel (Switzerland). Software-based questionnaires were used to collect data on employees’ age and sex, socio-economic and professional aspects, whether they were suffering from AD, PSO, chronic spontaneous urticaria (CSU), lichen (ruber) planus (LP), and/or HS as well as details regarding the disease course. Patients were also asked to state if their disease was diagnosed by a physician. Furthermore, patients were asked whether they were currently under medical care for their skin disease. The extent to which the patients consider their CISD to be controlled/treated and the impact of their CISD on their overall life were asked based on five answer categories (not at all/hardly/ moderately/well/completely and very severe/severe/moderate/little/not at all, respectively) (self-designed questionnaire, can be provided by corresponding authors upon request). For the assessment of the impairment of work ability and productivity and the general daily activity during the last 7 days due to respective CISD, the work productivity and activity impairment (WPAI) questionnaire was used. The WPAI is a validated six-question questionnaire (24). Scores range between 0 and 100%, with higher scores indicating greater impairment. The survey lasted from June 2022 to July 2022 (Germany) and July 2022 to August 2022 (Switzerland).

### 2.2 Statistical analysis

Statistical calculations were made in SPSS Statistics software (IBM), version 27. Kruskall-Wallis test, Pearson *Χ*^2^ test, and Spearman correlation test were applied as indicated. Global alpha was set at 5%.

## 3 Results

### 3.1 Characteristics of survey participants

To investigate CISD patients’ perception of the control of their skin disease and to identify aspects associated with poor control, the employees of a pharmaceutical company in the locations in Germany and Switzerland were asked to provide anonymous information via a software-based questionnaire. The questionnaire was sent out via email and newsletter to about 15.000 employees – concrete numbers on read-through rate are unknown. Overall, 905 employees answered the questionnaire. Of these, 222 participants (24.5%) reported having at least one of the following CISDs: AD, PSO, CSU, HS, and LP. Among them, 23 stated having two CISDs, and two and one stated having three and four CISDs, respectively, resulting in a total of 252 cases. The prevalence of the investigated CISDs were: AD: 12.0%, PSO: 7.6%, CSU: 3.8%, HS: 1.7%, and LP: 0.5%. Of the 222 people having stated to suffer from investigated CISDs, 195 delivered a detailed report about at least one skin disease (211 cases). Of these 211 cases, 85.3% were diagnosed by a physician according to patient statements. The characteristics of these 195 participants are presented in Supplementary Table S1.

### 3.2 Patients’ perception of the control of their skin disease

**Figure 1:**
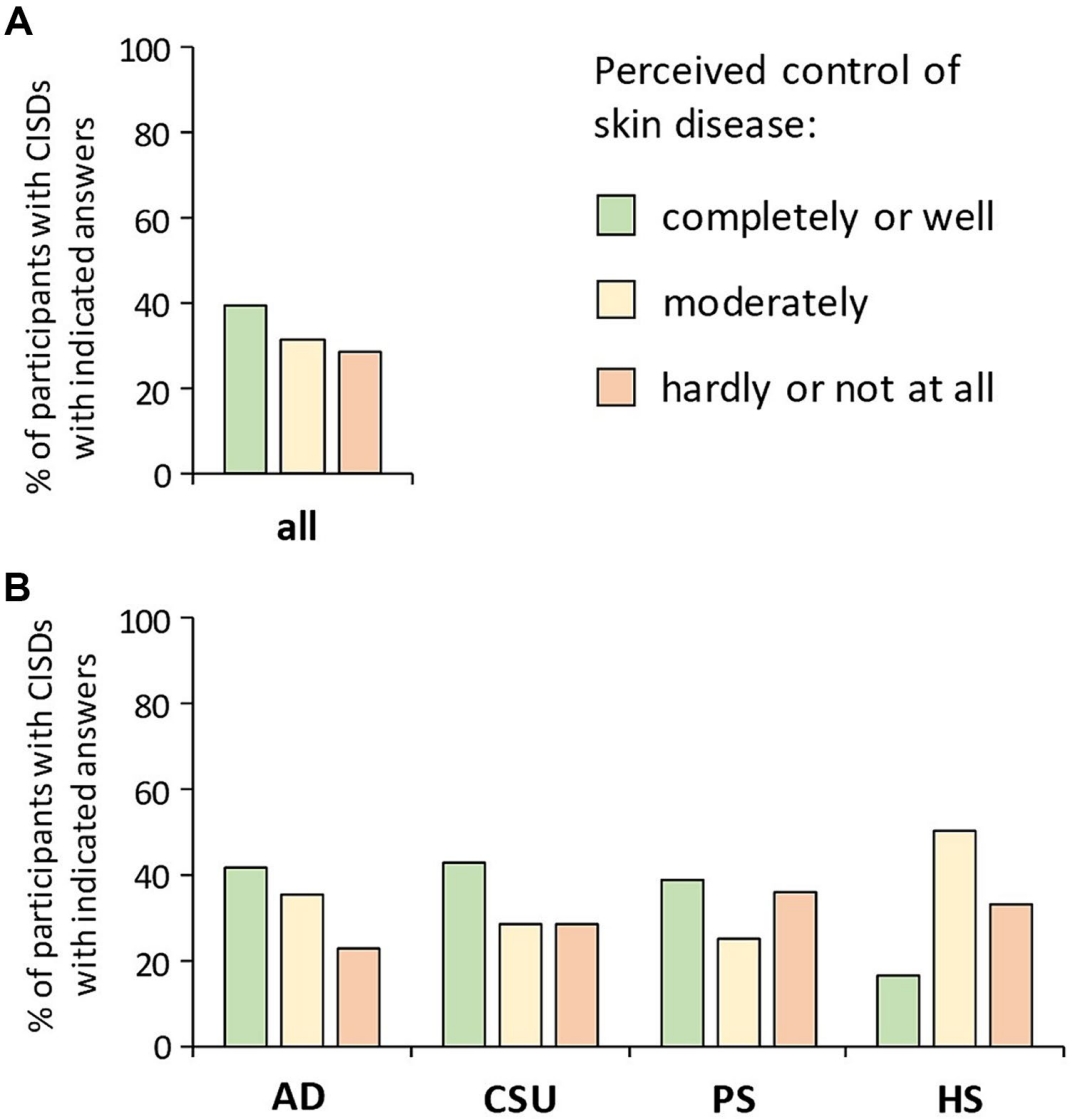
Patients’ perception of the control of their skin disease. Participants suffering from CISD were asked about how well they find their skin disease controlled. **(A)** The bar chart presents percentages of total cases (*n* = 202) with indicated answers. **(B)** The bar chart presents percentages of cases with indicated answers, broken down into the diseases most common among the participants [AD (*n* = 101), CSU (*n* = 28), PSO (*n* = 64), HS (*n* = 6)].

Participants suffering from CISD were asked about how well they consider their skin disease controlled. Figure 1A shows the proportions of participants considering their CISD as being: (i) completely or well controlled, (ii) moderately controlled, or (iii) hardly or not at all controlled. Only 39.6% described their skin disease as being completely or well controlled. The proportion of participants considering their CISD as being hardly or not at all controlled (in the following together referred to as poorly controlled) was 28.7%. When looking at the specific types of CISD, about one third of participants with PSO (35.9%) or HS (33.3%) reported poor disease control (Figure 1B). Participants suffering from CSU or AD showed the largest proportion of those reporting completely or well controlled CISD (42.9 and 41.6%, respectively), while this proportion was particularly small in case of HS (16.7%).

### 3.3 Possible reasons for patients’ perceived poor control of their skin disease

**Figure 2:**
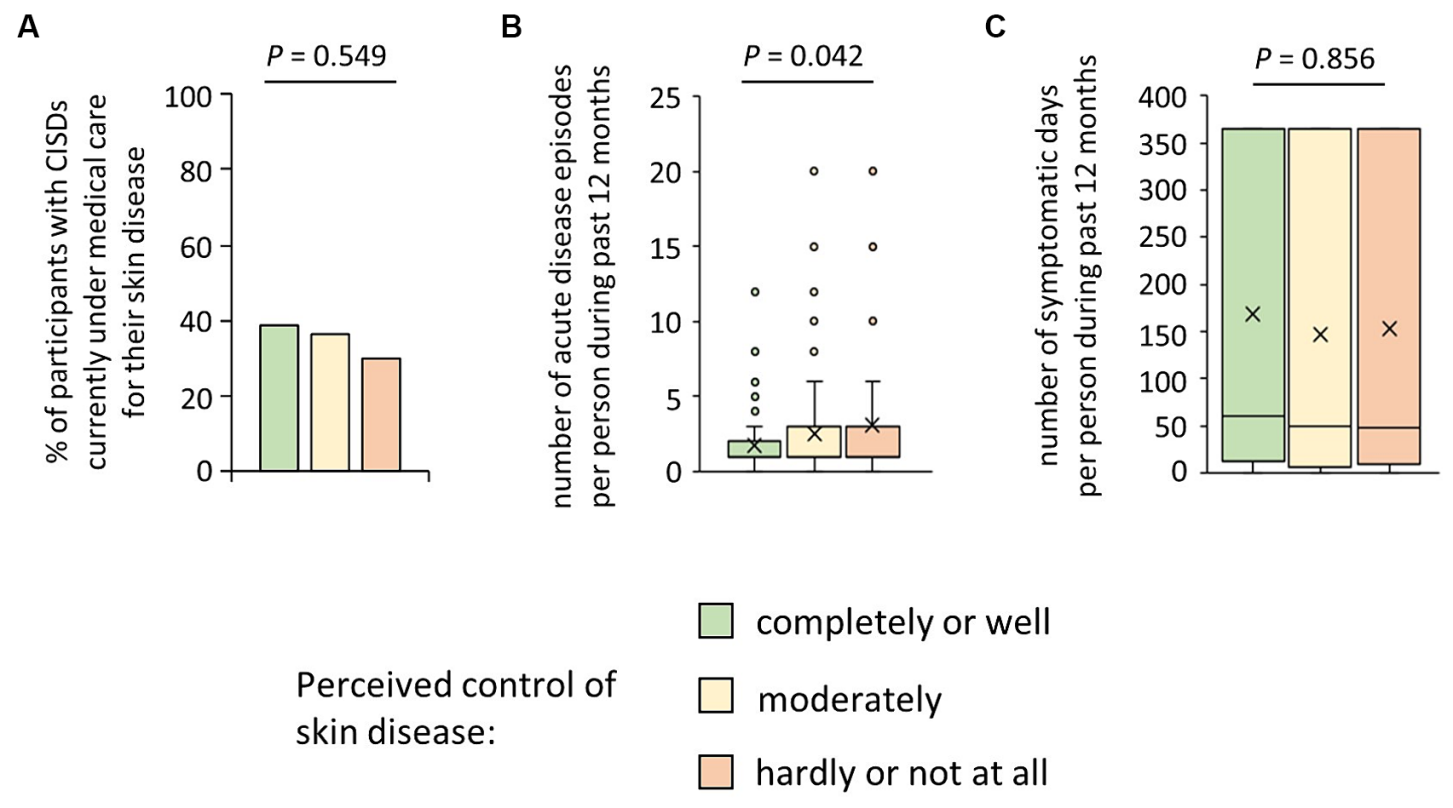
Possible reasons for patients’ perceived poor control of their skin disease. **(A)** Participants suffering from CISD were asked whether they were currently under medical care for their skin disease. The bar chart presents percentages of cases (*n* = 200) who answered the question with yes, broken down into their estimate of control of their skin disease. The *p*-value (Pearson *Χ*^2^ test) is presented. **(B)** Participants suffering from CISD were asked how many acute disease episodes they had in the last 12 months. Answers given by cases (*n* = 201), broken down into their estimate of control of their skin disease, are presented as box-and-whisker plots, with the maximum length of box whiskers corresponding to the most extreme values in the 1.5 fold interquartile range, outliers displayed as dots, and the “x” representing the mean of the data. The *p*-value (Kruskal Wallis test) is presented. **(C)** Participants suffering from CISD were asked how many acute disease episodes they had in the last 12 months and the average duration of these episodes, to calculate the total number of symptomatic days per year. Results (*n* = 201), broken down into their estimate of control of their skin disease, are presented as box-and-whisker plots, with the maximum length of box whiskers corresponding to the most extreme values in the 1.5 fold interquartile range, outliers displayed as dots, and the “x” representing the mean of the data. The *p*-value (Kruskal Wallis test) is presented.

We then wondered why such a substantial proportion of participants with CISDs considered their disease as poorly controlled and inquired whether they were currently under medical care for their skin disease. As shown in Figure 2A, only 35.5% stated that they were. Interestingly, this proportion did not vary between the three disease-control categories (*p* = 0.549). When asking about the CISD course in the last 12 months, we found a negative association between perceived disease control and the number of acute CISD phases (*p* = 0.042) (Figure 2B), which was confirmed by correlation testing (r_s_ = 0.16, *p* = 0.031). Surprisingly, there was no association between perceived disease control and the total number of symptomatic days per year (*p* = 0.856) (Figure 2C).

### 3.4 Possible consequences of patients’ perceived poor control of their skin diseases

**Figure 3:**
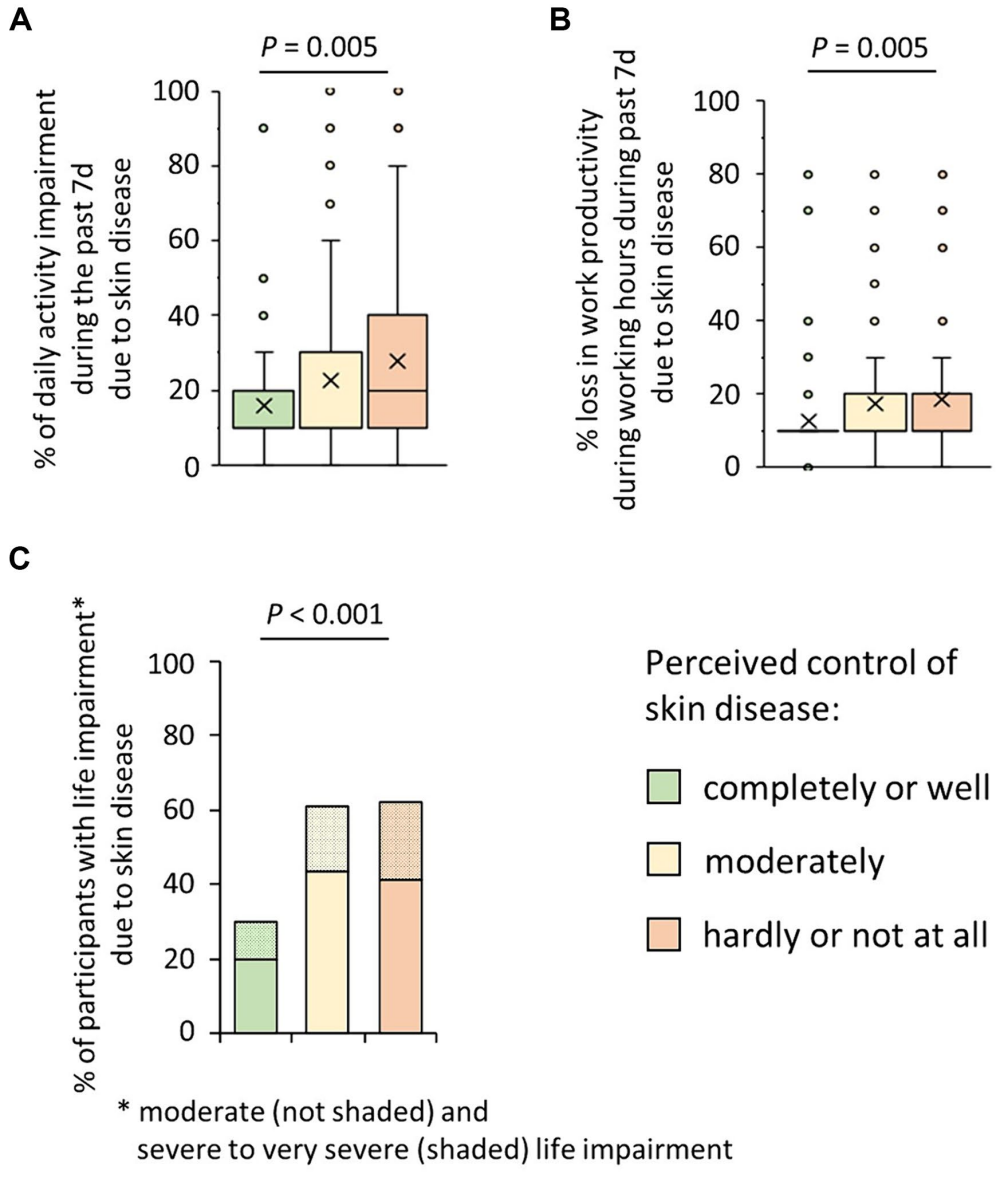
Possible consequences of patients’ perceived poor control of their skin disease. **(A)** Participants suffering from CISD were asked about their estimate of impairment of general daily activities (outside their professional activity) due to their skin disease in the past seven days (WPAI, on a 0–10 scale, corresponding to 0–100%). Answers given by the cases (*n* = 202), broken down into their estimate of control of their skin disease, are presented as box-and-whisker plots, with the maximum length of box whiskers corresponding to the most extreme values in the 1.5 fold interquartile range, outliers displayed as dots, and the “x” representing the mean of the data. The *p*-value (Kruskal Wallis test) is presented. **(B)** Participants suffering from CISD were asked about their estimate of impairment while working due to their skin disease in the past seven days (WPAI, presenteeism; on a 0–10 scale, corresponding to 0–100%). Answers given by the cases (*n* = 202), broken down into their estimate of control of their skin disease, are presented as box-and-whisker plots, with the maximum length of box whiskers corresponding to the most extreme values in the 1.5 fold interquartile range, outliers displayed as dots, and the “x” representing the mean of the data. The *p*-value (Kruskal Wallis test) is presented. **(C)** Participants suffering from CISD were asked how much their skin disease does impair their life. The bar chart presents percentages of cases (*n* = 202) who stated a moderate (not shaded) or a severe or very severe (shaded) life impairment, broken down into their estimate of control of their skin disease. *p*-value (Pearson *Χ*^2^ test) is presented.

Thirdly, we were interested in the effects of observed poor disease control on the general daily activity and the work productivity and ability of those affected. There was a clear positive association of poor disease control and the percent general daily activity impairment due to skin disease (mean ± SD; completely or well controlled disease: 16.2 ± 15.9% of general daily activity impairment; moderately controlled disease: 23.1 ± 23.9% of general daily activity impairment; hardly or not at all controlled disease: 28.3 ± 24.5% of general daily activity impairment; *p* = 0.005) (Figure 3A). Moreover, the percent loss in work productivity during working hours due to skin disease (presenteeism) increased dependent on the poorness of disease control (mean ± SD; completely or well controlled disease: 12.5 ± 12.1%; moderately controlled disease: 17.5 ± 17.0%; hardly or not at all controlled disease: 19.0 ± 15.9%; *p* = 0.005) (Figure 3B). Correlation analyses further confirmed the statistical link with both the general activity impairment (r_s_ = 0.24, *p* = 0.001) and presenteeism (r_s_ = 0.20, *p* = 0.001).

Finally, when asking the participants about the impact of the CISDs on their overall life, 41.4 and 20.7% of participants with poor disease control stated a moderate or severe to very severe impact (*P* = <0.001) (Figure 3C). Correlation analysis also demonstrated the association between lower of disease control and the negative impact of disease on the overall lives of those affected (r_s_ = 0.31, *p* < 0.001). Considering the type of skin diseases (CSU, AD, PSO, HS), participants with HS most often (33.3%) answered that the CISD had a severe or very severe impact on their lives (data not shown).

## 4 Discussion

### 4.1 Poor skin disease control and its consequences

Intensive work by basic scientists, clinical researchers, and pharmaceutical company employees over the past two decades has led to the approval of highly effective drugs for certain CISDs (e.g., PSO) (7). To our surprise, in the investigated health-aware environment, almost 30% of the participants with CISDs rated their skin disease as hardly or non-controlled. The proportion of participants feeling this way was particularly high among participants with PSO, for whom highly effective drugs have been approved (7), and patients with HS, who were only moderately treatable with drugs at the time of the survey (13). We found significant general daily activity impairment, relevant presenteeism, and negative impact on the life as a whole as possible consequences of the inadequate disease control. Poor skin disease control therefore seems to have a significant impact on both those affected and the society. We have not found any study comparable to our work (internal company survey). Instead, numerous surveys conducted in medical institutions have been published, which, however, may not fully represent the total patient population, since only patients in medical care are represented. Relevant data come from some general population-based studies conducted online. Using an online questionnaire, a study run in 2016 in France, Germany, Italy, Spain, and UK documented 24.7% presenteeism for AD patients (28). A Japanese study from 2017 showed dissatisfaction with their health (25.5, 28.7, and 31.7%) and high presenteeism (22.4, 15.4, and 21.2%) among patients with AD, PSO, and CU, respectively (29). The value of 16% presenteeism determined in our study for all participants with skin diseases is slightly lower than the presenteeism in the studies described above. This could be related to the location of the survey (in our case in a company). According to an online study from Ireland published in 2021, 52% of AD patients considered their disease as being poorly controlled (30).

### 4.2 Reasons for poor skin disease control

In contrast to the consequences, we found only vague hints for the reasons for the perceived poor disease control. There was an association with the number of acute disease phases, but not with the number of symptomatic days per year; thus, the disease course (phasic) certainly plays a role. Interestingly, no association with lacking current medical care was found, which was generally high.

We speculate that restrictions in medical care due to racial and ethnic aspects, which are relevant in other parts of the world (31), are not likely to have a relevant impact in our study. We rather suspect that the major cause for insufficient current medical care is a disappointment with medical care due to either delay in diagnosis [e.g., HS (32)], a limited efficacy of approved drugs [e.g., HS (13)], or the doctors’ hesitation to prescribe expensive, albeit highly effective drugs (33, 34). Regarding prescriptions, the most prevalent barriers to prescription of biologics reported by physicians in Germany in 2017 were the high cost, the low reimbursement and the fear of recourse (35). Unfortunately, this situation seems not to have relevantly changed (36). For example, only 3.7% of PSO patients were treated with biologics in Germany in 2020 (33). Data collected in ten western countries in 2019 showed that only 2.3% of patients with AD received biologics (37) and that in 75% of patients, treatment expectations were met only partially or not at all (37). With HS, the situation may be even worse. HS is a disease that, due to the specific pathomechanisms, leads to irreversible destruction of the normal skin architecture (38) and the classic treatment of HS does not improve patient’s quality of life in the long term (3). Patients consider a sustained therapeutic success as most important (39, 40) and treatment with biologics was associated with higher patients’ satisfaction (41). Nevertheless, data from Denmark indicated a mean time from the first systemic therapy to the start of a biological therapy of 15.3 years (42).

### 4.3 Study limitations

There are several limitations in our study. A relatively small proportion of employees answered the questions asked, which could create a potential bias. Moreover, the study design did not include assessment of skin disease severity and therapy recording by a dermatologist.

## 5 Conclusion

This study, unique in its focus on an environment with health-awareness and high socio-economic standard, highlights the inadequate disease control in individuals with CISDs, including psoriasis and HS. The correlation of the subjective perception of disease control by those affected with the disease flare frequency points to an actual existing issue, with limited efficacy of approved drugs and/or the physicians’ hesitation to prescribe highly effective, albeit costly, medications as potential contributors. The study further points out the substantial impact of inadequate CISD control on individuals and society.

## Data availability statement

Further data will be made available upon request according to the legal possibilities by the corresponding authors.

## Ethics statement

This survey was conducted in a blinded and voluntary way as patient-oriented program. All participants have agreed on the data collection and on possible publication of anonymous and aggregated data. The evaluations shown are secondary data evaluations of the original data sets. An ethics approval was not required for this study (Ethics commission of the Bavarian State Medical Association/Bayerischen Landesärztekammer, Germany).

## Author contributions

KWo: Visualization, Writing – original draft, Writing – review & editing. MS: Writing – review & editing, Conceptualization, Investigation. J-TM: Writing – review & editing, Conceptualization. FW: Writing – review & editing, Investigation, Project administration. KWa: Writing – review & editing, Investigation, Project administration. WF: Visualization, Writing – review & editing, Data curation, Formal analysis, Methodology. PN: Writing – review & editing, Conceptualization, Funding acquisition. RS: Writing – review & editing, Conceptualization, Formal analysis.

## References

[1] Richard MA Paul C Nijsten T Gisondi P Salavastru C Taieb C. Prevalence of Most common skin diseases in Europe: a population-based study. J Eur Acad Dermatol Venereol. (2022) 36:1088–96. doi: 10.1111/jdv.18050, PMID: 35274366 PMC9415115

[2] Sabat R Wolk K Loyal L Docke WD Ghoreschi K. T cell pathology in skin inflammation. Semin Immunopathol. (2019) 41:359–77. doi: 10.1007/s00281-019-00742-7, PMID: 31028434 PMC6505509

[3] Zhang B Roesner LM Traidl S Koeken V Xu CJ Werfel T. Single-cell profiles reveal distinctive immune response in atopic dermatitis in contrast to psoriasis. Allergy. (2023) 78:439–53. doi: 10.1111/all.15486, PMID: 35986602

[4] Langan SM Irvine AD Weidinger S. Atopic dermatitis. Lancet. (2020) 396:345–60. doi: 10.1016/S0140-6736(20)31286-1, PMID: 32738956

[5] Rojahn TB Vorstandlechner V Krausgruber T Bauer WM Alkon N Bangert C. Single-cell transcriptomics combined with interstitial fluid proteomics defines cell type-specific immune regulation in atopic dermatitis. J Allergy Clin Immunol. (2020) 146:1056–69. doi: 10.1016/j.jaci.2020.03.041, PMID: 32344053

[6] Wolk K Kunz S Witte E Friedrich M Asadullah K Sabat R. Il-22 increases the innate immunity of tissues. Immunity. (2004) 21:241–54. doi: 10.1016/j.immuni.2004.07.007, PMID: 15308104

[7] Ghoreschi K Balato A Enerback C Sabat R. Therapeutics targeting the Il-23 and Il-17 pathway in psoriasis. Lancet. (2021) 397:754–66. doi: 10.1016/S0140-6736(21)00184-7, PMID: 33515492

[8] Maeda K Tanioka T Takahashi R Watanabe H Sueki H Takimoto M. Mcam+Cd161-Th17 subset expressing Cd83 enhances Tc17 response in psoriasis. J Immunol. (2023) 210:1867–81. doi: 10.4049/jimmunol.2200530, PMID: 37186262

[9] Cole S Manghera A Burns L Barrett J Yager N Rhys H. Differential regulation of Il-17a and Il-17f via Stat5 contributes to psoriatic disease. J Allergy Clin Immunol. (2023) 152:783–98. doi: 10.1016/j.jaci.2023.03.035, PMID: 37244461

[10] Kolkhir P Gimenez-Arnau AM Kulthanan K Peter J Metz M Maurer M. Urticaria. Nat Rev Dis Primers. (2022) 8:61. doi: 10.1038/s41572-022-00389-z 36109590

[11] Cildir G Toubia J Yip KH Zhou M Pant H Hissaria P. Genome-wide analyses of chromatin state in human mast cells reveal molecular drivers and mediators of allergic and inflammatory diseases. Immunity. (2019) 51:949–65 e6. doi: 10.1016/j.immuni.2019.09.021, PMID: 31653482

[12] Rutter KJ Peake M Hawkshaw NJ Scholey R Bulfone-Paus S Friedmann PS. Solar Urticaria involves rapid mast cell Stat3 activation and neutrophil recruitment, with Fcepsilonri as an upstream regulator. J Allergy Clin Immunol. (In press) (2024). doi: 10.1016/j.jaci.2023.12.021, PMID: 38184075

[13] Sabat R Jemec GBE Matusiak L Kimball AB Prens E Wolk K. Hidradenitis Suppurativa. Nat Rev Dis Primers. (2020) 6:18. doi: 10.1038/s41572-020-0149-1 32165620

[14] Wolk K Warszawska K Hoeflich C Witte E Schneider-Burrus S Witte K. Deficiency of Il-22 contributes to a chronic inflammatory disease: Pathogenetic mechanisms in acne Inversa. J Immunol. (2011) 186:1228–39. doi: 10.4049/jimmunol.0903907, PMID: 21148041

[15] Wolk K Brembach TC Šimaitė D Bartnik E Cucinotta S Pokrywka A. Activity and components of the granulocyte Colony-stimulating factor pathway in hidradenitis Suppurativa. Br J Dermatol. (2021) 185:164–76. doi: 10.1111/bjd.19795, PMID: 33400270

[16] Sabat R Šimaitė D Gudjonsson JE Brembach TC Witte K Krause T. Neutrophilic granulocyte-derived B-cell activating factor supports B cells in skin lesions in hidradenitis Suppurativa. J Allergy Clin Immunol. (2023) 151:1015–26. doi: 10.1016/j.jaci.2022.10.034, PMID: 36481267

[17] Ujiie H Rosmarin D Schön MP Ständer S Boch K Metz M. Unmet medical needs in chronic, non-communicable inflammatory skin diseases. Front Med (Lausanne). (2022) 9:875492. doi: 10.3389/fmed.2022.875492, PMID: 35755063 PMC9218547

[18] Schneider-Burrus S Tsaousi A Barbus S Huss-Marp J Witte K Wolk K. Features associated with quality of life impairment in hidradenitis Suppurativa patients. Front Med (Lausanne). (2021) 8:676241. doi: 10.3389/fmed.2021.676241, PMID: 33987196 PMC8112201

[19] Thyssen JP Halling AS Schmid-Grendelmeier P Guttman-Yassky E Silverberg JI. Comorbidities of atopic dermatitis-what does the evidence say? J Allergy Clin Immunol. (2023) 151:1155–62. doi: 10.1016/j.jaci.2022.12.002, PMID: 36621338

[20] Enos CW Ramos VL McLean RR Lin TC Foster N Dube B. Cardiometabolic multimorbidity is common among patients with psoriasis and is associated with poorer outcomes compared to those without comorbidity. J Dermatolog Treat. (2022) 33:2975–82. doi: 10.1080/09546634.2022.2089329, PMID: 35737885

[21] Papapostolou N Xepapadaki P Katoulis A Makris M. Comorbidities of chronic Urticaria: a glimpse into a complex relationship. Front Allergy. (2022) 3:1008145. doi: 10.3389/falgy.2022.1008145 36465885 PMC9712803

[22] Woo YR Cho M Do Han K Cho SH Lee JH. Atopic dermatitis and the risk of myocardial infarction and all-cause mortality: a Nationwide population-based cohort study. Allergy Asthma Immunol Res. (2023) 15:636–46. doi: 10.4168/aair.2023.15.5.636, PMID: 37827980 PMC10570776

[23] Tiri H Jokelainen J Timonen M Tasanen K Huilaja L. Substantially reduced life expectancy in patients with hidradenitis Suppurativa: a Finnish Nationwide registry study. Br J Dermatol. (2019) 180:1543–4. doi: 10.1111/bjd.17578, PMID: 30597518

[24] Schneider-Burrus S Kalus S Fritz B Wolk K Gomis-Kleindienst S Sabat R. The impact of hidradenitis Suppurativa on professional life. Br J Dermatol. (2023) 188:122–30. doi: 10.1093/bjd/ljac027, PMID: 36689513

[25] Pandya A Adah E Jones B Chevalier R. The evolving landscape of immunotherapy for the treatment of allergic conditions. Clin Transl Sci. (2023) 16:1294–308. doi: 10.1111/cts.13546, PMID: 37170653 PMC10432873

[26] Sabat R Gudjonsson JE Brembilla NC van Straalen KR Wolk K. Biology of Interleukin-17 and novel therapies for hidradenitis Suppurativa. J Interf Cytokine Res. (2023) 43:544–56. doi: 10.1089/jir.2023.0105, PMID: 37824200

[27] Casale TB. Novel biologics for treatment of chronic spontaneous Urticaria. J Allergy Clin Immunol. (2022) 150:1256–9. doi: 10.1016/j.jaci.2022.06.027, PMID: 36180286

[28] Eckert L Gupta S Gadkari A Mahajan P Gelfand JM. Burden of illness in adults with atopic dermatitis: analysis of National Health and wellness survey data from France, Germany, Italy, Spain, and the United Kingdom. J Am Acad Dermatol. (2019) 81:187–95. doi: 10.1016/j.jaad.2019.03.037 30905805

[29] Itakura A Tani Y Kaneko N Hide M. Impact of chronic Urticaria on quality of life and work in Japan: results of a real-world study. J Dermatol. (2018) 45:963–70. doi: 10.1111/1346-8138.14502, PMID: 29897137 PMC6099381

[30] Murray G O'Kane M Watson R Tobin AM. Psychosocial burden and out-of-pocket costs in patients with atopic dermatitis in Ireland. Clin Exp Dermatol. (2021) 46:157–61. doi: 10.1111/ced.14422, PMID: 32803784

[31] Nock MR Barbieri JS Krueger LD Cohen JM. Racial and ethnic differences in barriers to care among us adults with chronic inflammatory skin diseases: a cross-sectional study of the all of us research program. J Am Acad Dermatol. (2023) 88:568–76. doi: 10.1016/j.jaad.2022.09.054 36244557

[32] Kokolakis G Wolk K Schneider-Burrus S Kalus S Barbus S Gomis-Kleindienst S. Delayed diagnosis of hidradenitis Suppurativa and its effect on patients and healthcare system. Dermatology. (2020) 236:421–30. doi: 10.1159/000508787, PMID: 32610312 PMC7592906

[33] Heidbrede T Mevius A Kessel S Wilke T Maywald U Thiem A. Real-world systemic treatment of patients with psoriasis: a retrospective study based on German claims data. J Dtsch Dermatol Ges. (2023) 21:611–9. doi: 10.1111/ddg.15030_g, PMID: 37073599

[34] Schild M Weber V Thaçi D Kisser A Galetzka W Enders D. Treatment patterns and healthcare resource utilization among patients with atopic dermatitis: a retrospective cohort study using German health claims data. Dermatol Ther (Heidelb). (2022) 12:1925–45. doi: 10.1007/s13555-022-00773-3, PMID: 35871680 PMC9357591

[35] Schielein MC Tizek L Rotter M Konstantinow A Biedermann T Zink A. Guideline-compliant prescription of biologicals and possible barriers in dermatological practices in Bavaria. J Eur Acad Dermatol Venereol. (2018) 32:978–84. doi: 10.1111/jdv.14811, PMID: 29356181

[36] Weiss D Nordhorn I Tizek L Werfel T Zink A Biedermann T. Prescription behaviour and barriers to prescription of biologicals for treatment of chronic inflammatory skin diseases in dermatological practice in two German Federal States. Acta Derm Venereol. (2021) 101:adv00560. doi: 10.2340/00015555-3901, PMID: 34427313 PMC9425602

[37] Augustin M Costanzo A Pink A Seneschal J Schuster C Mert C. Real-world treatment patterns and treatment benefits among adult patients with atopic dermatitis: results from the atopic dermatitis patient satisfaction and unmet need survey. Acta Derm Venereol. (2022) 102:adv00830. doi: 10.2340/actadv.v102.3932, PMID: 36479885 PMC10508272

[38] Wolk K Join-Lambert O Sabat R. Aetiology and pathogenesis of hidradenitis Suppurativa. Br J Dermatol. (2020) 183:999–1010. doi: 10.1111/bjd.19556, PMID: 33048349

[39] Willems D Hiligsmann M van der Zee HH Sayed CJ Evers S. Identifying unmet care needs and important treatment attributes in the Management of Hidradenitis Suppurativa: a qualitative interview study. Patient. (2022) 15:207–18. doi: 10.1007/s40271-021-00539-7, PMID: 34368925 PMC8349666

[40] Faverio K Peitsch WK Gorig T Schneider-Burrus S Benzel F Goebeler M. Patient preferences in hidradenitis Suppurativa (approach-Hs): a discrete choice experiment. J Dtsch Dermatol Ges. (2022) 20:1441–52. doi: 10.1111/ddg.14886_g, PMID: 36321358

[41] Midgette B Strunk A Akilov O Alavi A Ardon C Bechara FG. Factors associated with treatment satisfaction in patients with hidradenitis Suppurativa: results from the global voice project. Br J Dermatol. (2022) 187:927–35. doi: 10.1111/bjd.21798, PMID: 36056741

[42] Ring HC Yao Y Maul JT Ingram JR Frew JW Thorsen J. The road to biologics in patients with hidradenitis Suppurativa: a Nationwide drug utilization study. Br J Dermatol. (2022) 187:523–30. doi: 10.1111/bjd.21673, PMID: 35603888 PMC9796665

