# Supplementary material for: Patient-reported assessment of medical care for chronic inflammatory skin diseases: an enterprise-based survey
Source: Front Med (Lausanne). 2024 Apr 18;11:1384055. doi: 10.3389/fmed.2024.1384055 (PMC11064793; doi:10.3389/fmed.2024.1384055)
Supplement: Supplementary file 1 [file Table_1.DOCX]

Supplementary Material

# Supplementary Tables

**Table S1: Characteristics of participants who delivered a detailed report about at least one skin disease (n=195).**

| **Characteristic** | **Percentage** |
| --- | --- |
| **Age** | |
| <20 years old | 1 % |
| 20-29 years old | 9 % |
| 30-39 years old | 23 % |
| 40-49 years old | 38 % |
| ≥50 years old | 29 % |
| **Sex** | |
| female | 64 % |
| male | 35 % |
| no indication | 1 % |
| **Company location** | |
| Germany | 18.5 % |
| Switzerland | 81.5 % |
| **Working hours per week** | |
| <38 hours | 78 % |
| 30-38 hours | 13 % |
| 20-29 hours | 7 % |
| <20 hours | 1 % |
| no indication | 1 % |
|  | |
